# Acquisition of pluripotency in the chick embryo occurs during intrauterine embryonic development via a unique transcriptional network

**DOI:** 10.1186/s40104-018-0246-0

**Published:** 2018-04-10

**Authors:** Jae Yong Han, Hyo Gun Lee, Young Hyun Park, Young Sun Hwang, Sang Kyung Kim, Deivendran Rengaraj, Byung Wook Cho, Jeong Mook Lim

**Affiliations:** 1 Department of Agricultural Biotechnology and Research Institute of Agriculture and Life Sciences, College of Agriculture and Life Sciences, Seoul National University, Seoul, 08826 Korea; 2 Institute for Biomedical Sciences, Shinshu University, Minamiminowa, Nagano, 399-4598 Japan; 3 Department of Animal Science and Technology, Chung-Ang University, Anseong, Gyeonggi-do 17546 Korea; 4 Department of Animal Science, College of Natural Resources and Life Sciences, Pusan National University, Miryang, 50463 Korea

**Keywords:** Avian, Embryonic development, NANOG, Pluripotency, Transcriptional factor

## Abstract

**Background:**

Acquisition of pluripotency by transcriptional regulatory factors is an initial developmental event that is required for regulation of cell fate and lineage specification during early embryonic development. The evolutionarily conserved core transcriptional factors regulating the pluripotency network in fishes, amphibians, and mammals have been elucidated. There are also species-specific maternally inherited transcriptional factors and their intricate transcriptional networks important in the acquisition of pluripotency. In avian species, however, the core transcriptional network that governs the acquisition of pluripotency during early embryonic development is not well understood.

**Results:**

We found that chicken *NANOG* (*cNANOG*) was expressed in the stages between the pre-ovulatory follicle and oocyte and was continuously detected in Eyal-Giladi and Kochav stage I (EGK.I) to X. However, *cPOUV* was not expressed during folliculogenesis, but began to be detectable between EGK.V and VI. Unexpectedly, *cSOX2* could not be detected during folliculogenesis and intrauterine embryonic development. Instead of *cSOX2*, *cSOX3* was maternally inherited and continuously expressed during chicken intrauterine development. In addition, we found that the pluripotency-related genes such as *cENS-1*, *cKIT*, *cLIN28A*, *cMYC*, *cPRDM14*, and *cSALL4* began to be dramatically upregulated between EGK.VI and VIII.

**Conclusion:**

These results suggest that chickens have a unique pluripotent circuitry since maternally inherited c*NANOG* and *cSOX3* may play an important role in the initial acquisition of pluripotency. Moreover, the acquisition of pluripotency in chicken embryos occurs at around EGK.VI to VIII.

## Background

The acquisition of pluripotency plays a pivotal role in determining developmental fate during early vertebrate embryogenesis. In mammals, *Oct4*, *Sox2*, and *Nanog*, called core pluripotency transcription factors, act as specific modulators of pluripotency and can control the developmental fate of cells by inhibiting cellular differentiation [1, 2]. In mice, *Oct3/4* and *Sox2* are maternally inherited transcription factors [3], while *Nanog* is initially expressed in the compacted morulae [4]. These transcription factors collaborate to constitute a regulatory network, and share many target genes [5, 6]. They are expressed in every cell during the cleavage period, but are gradually restricted to the inner cell mass. Meanwhile, *Oct25* (*Pou5f3.2*), *Oct60* (*Pou5f3.3*), and *Sox3* are maternally inherited to establish the pluripotency network and initiate the maternal to zygotic transition in Xenopus. Since there is no ortholog of *Nanog* in Xenopus, *Ventx* plays an important role in pluripotency [7, 8]. In addition, in zebrafish, *Oct4* (*Pou5f3*), *SoxB1*, and *Nanog* are maternally inherited to establish the pluripotency network for zygotic genome activation (ZGA) [9, 10]. Although maternally inherited core transcription factors for the initial acquisition and organization of pluripotency are unique to each vertebrate species, their network in vertebrates is well conserved. However, the acquisition of pluripotency and the core pluripotency circuitry during early embryonic development has yet to be investigated in detail in birds. It has been reported that the transition from totipotent state to pluripotent state during early embryonic development seem to be accompanied by the pluripotency regulatory genes under core transcriptional network [11–14]. However, the intricate changes of transcriptional network under regulation of core pluripotency circuitry during the acquisition of pluripotency in avian species are not clear.

After fertilization, chicken embryos undergo a series of developmental events in utero for approximately 24 h, including cellularization, the ZGA and layers increase during the cleavage period, and lineage specification and layer reduction during area pellucida formation [15]. During chicken intrauterine development, the expression of core regulatory genes is spatiotemporally triggered or suppressed under tight transcriptional regulation. Such early developmental pathways, including ZGA, pluripotency acquisition, and lineage segregation, are systematic processes, governed by the concerted action of multiple unknown transcriptional networks [16–18]. In this regard, the core pluripotency transcription factors governing the acquisition of pluripotency with respect to developmental processes during chicken intrauterine development require further investigation. Here, for the first time, we examined the detailed spatiotemporal expression profiles of core pluripotency transcription factors, including chicken *NANOG* (*cNANOG)*, *POUV* (*cPOUV*) and *SOXB1* members (*cSOX2* and *cSOX3*), and determined the developmental stage for the acquisition of pluripotency during intrauterine embryonic development in chicken.

## Methods

### Experimental animals and animal care

The care and experimental use of chickens was approved by the Institute of Laboratory Animal Resources, Seoul National University (SNU-150827-1). Chickens were maintained according to a standard management program at the University Animal Farm, Seoul National University, Korea. The procedures for animal management, reproduction, and embryo manipulation were in adherence with the standard operating protocols of our laboratory.

### Alignment and conservation of protein sequences

In order to identify the percent identities of chicken NANOG, POUV, SOX2, and SOX3 proteins/functional domains with candidate vertebrate species, the NANOG, POUV, SOX2, and SOX3 amino acid sequences from *Gallus gallus*, *Homo sapiens*, *Mus musculus*, *Danio rerio*, and *Xenopus laevis* were aligned with Geneious software version 6.0 (Biomatters, Auckland, New Zealand). Sequence information was obtained from the National Center for Biotechnology Information (NCBI) database (Table 1). All protein sequences were aligned using the Blosum62 scoring matrix, with the gap open penalty set at 12 and the gap extension penalty set at 3.

**Table 1:** Protein sequence alignment of chicken NANOG, POUV, SOX2, and SOX3 with candidate vertebrate species

Protein	Species	Accession no.	Protein length	Percent identities of proteins^a^	Percent identities of functional domains^a^
cNANOG	*Gallus gallus*	NP_001139614	309	NA	Homeodomain
	*Homo sapiens*	NP_079141	305	26.0%	64.8%
	*Mus musculus*	NP_082292	305	27.5%	66.7%
	*Danio rerio*	AEZ64150	384	20.1%	61.1%
cPOUV	*Gallus gallus*	NP_001296301	389	NA	Homeodomain
	*Homo sapiens*	NP_002692	360	36.1%	66.7%
	*Mus musculus*	NP_038661	352	35.7%	66.7%
	*Danio rerio*	NP_571187	472	39.5%	64.8%
	*Xenopus laevis*	NP_001081342	445	37.1%	74.1%
cSOX2	*Gallus gallus*	AAB09662	315	NA	HMG domain
	*Homo sapiens*	NP_003097	317	93.4%	98.6%
	*Mus musculus*	NP_035573	319	92.2%	98.6%
	*Danio rerio*	NP_998283	315	90.2%	97.2%
	*Xenopus laevis*	NP_001081691	311	91.1%	100%
cSOX3	*Gallus gallus*	NP_989526	316	NA	HMG domain
	*Homo sapiens*	NP_005625	446	69.0%	97.2%
	*Mus musculus*	NP_033263	450	69.3%	97.2%
	*Danio rerio*	NP_001001811	300	79.0%	95.8%
	*Xenopus laevis*	NP_001007502	307	82.2%	98.6%

^a^Percent identities of chicken proteins or domains with other vertebrate species

### Collection of intrauterine eggs, follicles, and oocytes from hens

The intrauterine embryonic developmental period in the chicken is divided into 10 stages, described and named by Eyal-Giladi and Kochav, and designated EGK.I through to EGK.X [19]. Intrauterine eggs were retrieved from White Leghorn (WL) hens by an abdominal massage technique from our earlier study [17]. Briefly, the abdomen was pushed gently until the shell gland was exposed. The surface of the shell gland expanded when an egg was located there for eggshell formation. After this expansion of the shell gland, the intrauterine egg was gently moved toward the cloaca via massage until it was released. Intrauterine blastoderms were classified according to the criteria of Eyal-Giladi and Kochav [19, 20]. The harvested blastoderms were fixed in 4% paraformaldehyde in phosphate-buffered saline (PBS) for subsequent experiments. Fertility and abnormalities in the collected blastoderms were determined according to morphology. For the collection of follicles and oocytes, WL hens were sacrificed and ovaries were collected. Follicles were categorized into F1 (30–35 mm), F3 (20–25 mm), F5 (10–15 mm), small yellow follicle (5–8 mm), and large white follicle (WF, 2–4 mm) [21, 22]. Follicles were dissected to separate theca and granulosa layers and were subsequently homogenized for isolation of RNA after washing with PBS.

### Reverse transcription-polymerase chain reaction (RT-PCR) and quantitative real-time PCR (qRT-PCR)

The total RNA of samples was extracted from pre-ovulatory follicles and intrauterine chicken embryos using TRIzol reagent (Invitrogen, Thermo Fisher Scientific, Carlsbad, CA, USA) according to the manufacturer’s instructions. The oviposited chicken embryos were classified according to the staging by Hamburger and Hamilton (HH) [23]. From the HH 26–28 embryos, RNA was extracted from chicken embryonic fibroblasts (CEFs) and intact primordial germ cells (PGCs) [24]. The complementary DNA (cDNA) of the sample was synthesized using the Superscript III First-strand Synthesis System (Invitrogen) according to the manufacturer’s protocol. The RT-PCR reaction mixture contained 2 μL of PCR buffer, 0.5 μL of 10 mmol/L dNTP mixture (Solgent, Daejeon, Korea), 10 pmoles each of forward and reverse primers (Table 2), 1 μL of cDNA and 1 IU of Taq DNA polymerase in a 20 μL final volume. RT-PCR was performed with an initial incubation at 95 °C for 10 min, followed by 30 cycles of 95 °C for 30 s, 60 °C for 30 s, and 72 °C for 30 s. PCR was terminated by a final incubation at 72 °C for 5 min. qRT-PCR was performed using the CFX96 Real-Time PCR Detection System (Bio-Rad, Hercules, CA, USA). The PCR reaction mixture contained 2 μL of PCR buffer, 0.5 μL of 10 mmol/L dNTP mixture (Solgent), 10 pmoles each of the forward and reverse primers (Table 2), 1 μL of cDNA, 1 μL of EvaGreen (Biotium, Fremont, CA, USA), and 1 IU of Taq DNA polymerase in a 20-μL final volume. qRT-PCR was performed with an initial incubation at 95 °C for 10 min, followed by 40 cycles of 95 °C for 30 s, 60 °C for 30 s, and 72 °C for 30 s. The reaction was terminated by a final incubation at the dissociation temperature. The relative gene expression was calculated after normalization with *GAPDH* and values at stage EGK.X using the formula 2^-ΔΔCt^ [25].

**Table 2:** Primer sequences used for RT-PCR, in situ hybridization, and qRT-PCR

Gene	Accession no.	Forward (5′ → 3′)	Reverse (5′ → 3′)	Amplicon size, bp
RT-PCR or in situ hybridization				
*cNANOG*	NM_001146142	CAGCAGACCTCTCCTTGACC	AAGCCCTCATCCTCCACAGC	586
*cPOUV*	NM_001309372	GCCAAGGACCTCAAGCACAA	ATGTCACTGGGATGGGCAGA	511
*cSOX2*	NM_205188	CACAACTCGGAGATCAGCAA	GTAGGTAGGCGATCCGTTCA	471
*cSOX3*	NM_204195	CGGCACCGTACCACTAACTC	GACTCGGAAGCGAACAAAAC	302
*cGAPDH*	NM_204305	CACAGCCACACAGAAGACGG	CCATCAAGTCCACAACACGG	443
qRT-PCR				
*cGAPDH*	NM_204305	ACACAGAAGACGGTGGATGG	GGCAGGTCAGGTCAACAACA	193
*cNANOG*	NM_001146142	CAGCAGACCTCTCCTTGACC	AAAAGTGGGGCGGTGAGATG	187
*cPOUV*	NM_001309372	TGAAGGGAACGCTGGAGAGC	ATGTCACTGGGATGGGCAGAC	231
*cSOX3*	NM_204195	CGGCACCGTACCACTAACTC	GACTCGGAAGCGAACAAAAC	302
*cENS-1*	NM_001080873	TGCTCGGCCTTCTGTATCAG	TTCCTCTCGGAACTCCACAG	181
*cTFCP2L1*	XM_422087	TCAGCACATTAAAAGCTGAAAGCA	AGCAATCTCAGTGAGGCACTA	110
*cTBX3*	NM_001270878	GTGGAAGACGACCCGAAAGT	CACCATCTCCGTGCCTCTTT	78
*cPRDM14*	XM_015282907	AAATTCCCCTGCCACCTCTG	CCCGCATGTGTTTGTTCAGG	154
*cKIT*	NM_204361	AGCGAACTTCACCTTACCCG	CTGGGAATCCAGTTGCCACA	181
*cLIN28A*	NM_001031774	CCGAGAATGAGTCCCAACCC	GGTGAATTCAACGGCTTCGC	197
*cMYC*	NM_001030952	GAGGAGAACGACAAGAGGCG	CACGCAGGGCAAAGAAACTC	85
*cSALL4*	NM_001080872	AATTCTGCCAGACGGGGAAG	GCTATGCCATTGCTGAGCAC	170

### In situ hybridization

To prepare hybridization probes, total RNA from each blastodermal stage was reverse transcribed, and the cDNA was amplified using the primers shown in Table 2. The PCR products of the correct size were cloned with the pGEM-T Easy Vector System (Promega, Madison, WI, USA). After sequence verification, the recombinant plasmids containing the genes of interest were amplified with T7 (T7: 5′-TGTAATACGACTCACTATAGGG-3′) and SP6-specific primers (SP6: 5′-CTATTTAGGTGACACTATAGAAT-3′) to prepare the templates for labeling with hybridization probes. Digoxigenin (DIG)-labeled sense and antisense hybridization probes of each gene were transcribed in vitro using the DIG RNA Labeling Kit (Roche Diagnostics, Basel, Switzerland). Whole mount in situ hybridization was performed following the standard protocol for chickens [26, 27]. In addition, intrauterine blastoderms were embedded in paraffin and sectioned at 10 μm on a HM 355S automatic microtome (Thermo Fisher Scientific). After deparaffinization, rehydration, and antigen retrieval, each slide was mounted with Vectashield Antifade Mounting Medium with DAPI (Vector Laboratories, Burlingame, CA, USA). The embryonic nuclei were evaluated under a Ti-U fluorescence microscope (Nikon, Tokyo, Japan).

### Statistical analysis

All data of qRT-PCR are expressed as mean ± standard error of mean from three independent experiments. GraphPad Prism software (GraphPad Software, La Jolla, CA, USA) was used to evaluate the data. Significant differences were evaluated by one-way ANOVA with Bonferroni’s multiple comparison test between developmental stages. *P* < 0.05 was considered statistically significant.

## Results

### Expression profiling of core pluripotent transcriptional factors during chicken intrauterine development

To investigate the conservation of transcriptional regulatory networks of pluripotency among vertebrates, initially, we compared protein identities and the conservation of core pluripotent transcription factors, including cPOUV, cSOX2, and cNANOG with human, mouse, zebrafish, and frog. As shown in Table 1, the identities of cNANOG and its homeodomain with the candidate vertebrate species was about 20–26% and 61–66%, respectively. The identities of cPOUV and its homeodomain with the candidate vertebrate species was about 35–39% and 64–74%, respectively. Interestingly, the identities of cSOX2 and its high mobility group (HMG) domain with the candidate vertebrate species was about 90–93% and 97–100%, respectively.

**Fig. 1:**
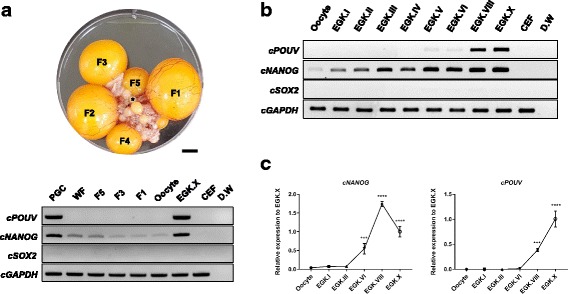
Core pluripotent transcription factors expression during folliculogenesis and intrauterine embryonic development in chicken. **a** RT-PCR was conducted to examine the maternally inherited pluripotent transcriptional factors, including *cPOUV*, *cNANOG*, and *cSOX2* during chick ovarian follicle development. Follicles in the ovary are indicated by hierarchical follicle (F1 to F5) and a representative white follicle is indicated by an asterisk. **b** RT-PCR was conducted to examine expression profiling of *cPOUV*, *cNANOG*, and *cSOX2* from the oocyte to EGK.X. CEF was used as a negative control for both A and B. **c** The *cPOUV* and *cNANOG* gene expression in oocyte and intrauterine chicken embryos relative to EGK.X was analyzed using qRT-PCR. *cGAPDH* was used as a reference gene. Results are shown as mean ± standard error of mean (*n* = 3). Significant differences of the relative gene expression between consecutive developmental stages (Oocyte vs. EGK.I, EGK.I vs. EGK.III, EGK.III vs. EGK.VI, EGK.VI vs. EGK.VIII, and EGK.VIII vs. EGK.X) were represented as *** *P* < 0.001 and **** *P* < 0.0001. Scale bar = 1 cm

To examine which of the core pluripotent transcription factors are maternally inherited in chicken, *cPOUV*, *cNANOG*, and *cSOX2* genes were evaluated on the stages between WFs and oocytes together with PGC, EGK.X, and CEF samples using RT-PCR. As shown in Fig. 1a, *cNANOG* was only expressed during folliculogenesis, indicating that *cNANOG* is maternally derived. *cPOUV* expression was only detected in PGCs and EGK.X embryo, and *cSOX2* expression was not detected in any of these samples. To understand the temporal regulation of pluripotency networks during chicken intrauterine development, we examined the expression profiles of core pluripotency transcription factors across developmental stages from the oocyte to the EGK.X embryo (Fig. 1b and c). The results of RT-PCR showed that *cNANOG* was detected continuously from the oocyte to stage EGK.X but *cPOUV* was first detectable at EGK.V and its expression was upregulated thereafter. Unexpectedly, *cSOX2* was not expressed during chicken intrauterine stages despite its important function in the pluripotency circuitry (Fig. 1b). The results of qRT-PCR showed highly correlative manner of *cPOUV* and *cNANOG* expressions in the samples examined (Fig. 1c). Taken together, these results suggest that *cNANOG* and *cPOUV*, but not *cSOX2*, are involved in the acquisition of pluripotency during early development in the chicken.

### Cellular localization of *cNANOG* and *cPOUV* from the oocyte to stage EGK.X

**Fig. 2:**
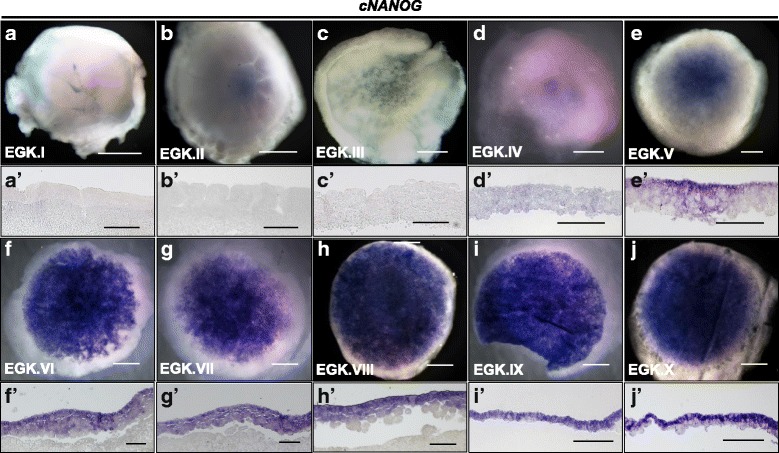
Cellular localization of *cNANOG* during intrauterine development of the chicken embryo. In situ hybridization was performed on the whole-mount (**a**–**j**) and longitudinal sections (**a’**–**j’**) of intrauterine chicken embryos to detect cellular localization of *cNANOG*. Scale bars = 1 mm (**a**–**j**) and 200 μm (**a’**–**j’**)

**Fig. 3:**
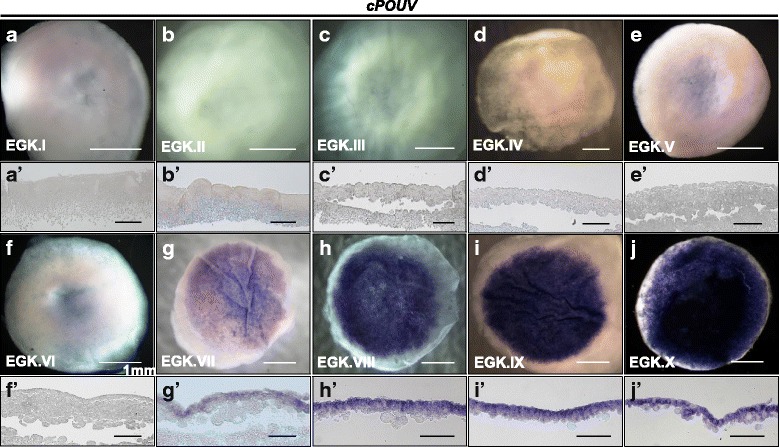
Cellular localization of *cPOUV* during intrauterine development of the chicken embryo. In situ hybridization was performed on the whole-mount (**a**–**j**) and longitudinal sections (**a’**–**j’**) of intrauterine chicken embryos to detect cellular localization of *cPOUV*. Scale bars = 1 mm (**a**–**j**) and 200 μm (**a’**–**j’**)

To determine the cellular localization and temporal expression of *cNANOG* and *cPOUV*, we conducted whole-mount in situ hybridization and longitudinal sections over the course of development from EGK.I to EGK.X. As shown in Fig. 2, *cNANOG* transcripts were rarely detected between EGK.I and EGK.III (Fig. 2a–c and a’–c’) and began to be detectable at EGK.IV (Fig. 2d and d’), where they were localized in a heterogeneous manner in the central region of the blastoderm (Fig. 2e and e’), and the intensity remarkably increased at stage EGK.VI (Fig. 2f and f’). During the period of area pellucida formation (EGK.VII–EGK.X), the *cNANOG* transcripts increased, and were localized to the upper layer of the blastoderm (Fig. 2g–j and g’–j’). At EGK.X, *cNANOG* transcripts were exclusively expressed in the epiblast region (Fig. 2j and j’). Meanwhile, *cPOUV* transcripts were not detected during the EGK.I–VI stages, at which point there is a period of cell layer increase (Fig. 3a–f and a’–f’). *cPOUV* transcripts started to be detected at EGK.VII and were clearly expressed in a salt-and-pepper manner in the blastoderm before EGK.X (Fig. 3g–j and g’–j’). At EGK.X, *cPOUV* transcripts were evenly expressed in the upper layer, called the epiblast, or expressed in a heterogeneous manner in the lower layer, called the hypoblast (Fig. 3j’).

### Expression profiling of *cSOX2* and *cSOX3* during intrauterine development

**Fig. 4:**
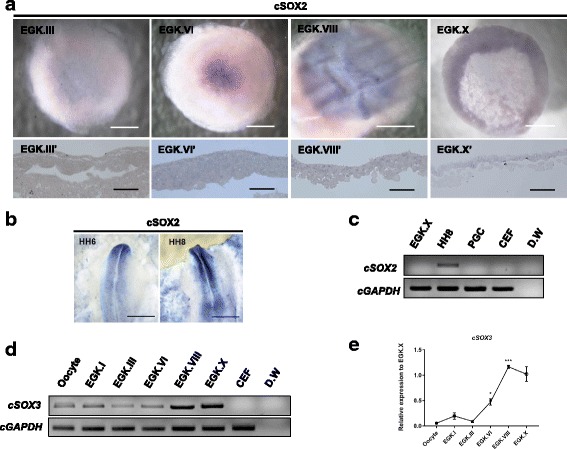
Expression profiling of *cSOX2* and *cSOX3* during chicken embryonic development. **a** In situ hybridization was performed on the whole-mount and longitudinal sections in pre-oviposition stages of chick blastoderm (EGK.III, EGK.VIII and EGK.X) to detect *cSOX2* transcripts. **b** *cSOX2* transcripts are clearly detected at neural induction stages between HH 6 and 8 by whole-mount in situ hybridization. **c** RT-PCR was conducted to confirm *cSOX2* expression in EGK.X, HH8, PGC, and CEF. **d** RT-PCR was conducted to examine *cSOX3* expression from the oocyte to the EGK.X embryo. CEF was used as a negative control. **e** The *cSOX3* gene expression in oocyte and intrauterine chicken embryos relative to EGK.X was analyzed using qRT-PCR. *cGAPDH* was used as a reference gene. Results are shown as mean ± standard error of mean (*n* = 3). Significant differences of the relative gene expression between consecutive developmental stages (Oocyte vs. EGK.I, EGK.I vs. EGK.III, EGK.III vs. EGK.VI, EGK.VI vs. EGK.VIII, and EGK.VIII vs. EGK.X) were represented as *P < 0.05 and *** *P* < 0.001. Scale bars = 1 mm (whole-mount of embryo) and 200 μm (longitudinal sections of embryo)

Since *cSOX2* was not detected in any of the samples tested by RT-PCR (Fig. 1), we further examined whether *cSOX2* is expressed during early embryonic development in chicken using whole-mount in situ hybridization. As shown in Fig. 4, *cSOX2* was not detected at the pre-ovipositional stages of the chicken embryo (Fig. 4a). Meanwhile, we confirmed that *cSOX2* was strongly expressed in the primitive streak at HH stages 6 and 8 (Fig. 4b and c). In addition, we examined the expression profiling of *cSOX3* (another member of SOXB1 family) during selective intrauterine development using RT-PCR and qRT-PCR. As shown in Fig. 4d, *cSOX3* was maternally inherited and continuously detected from the oocyte to the EGK.X embryo. As determined by qRT-PCR, *cSOX3* expression was sharply elevated after EGK.III (Fig. 4e). When we examine the identities of cSOX3 and its HMG domain with the candidate vertebrate species, it shows about 69–82% and 95–98% identities, respectively (Table 1). Moreover, similar to *cSOX2*, *cSOX1* expression was not detected in any stages during intrauterine development (data not shown). Collectively, these results imply that the process of pluripotency acquisition in chickens may be initiated by *cNANOG* and *cSOX3* ahead of *cPOUV* without *cSOX2*.

### Pluripotency-related marker expression during chicken intrauterine development

**Fig. 5:**
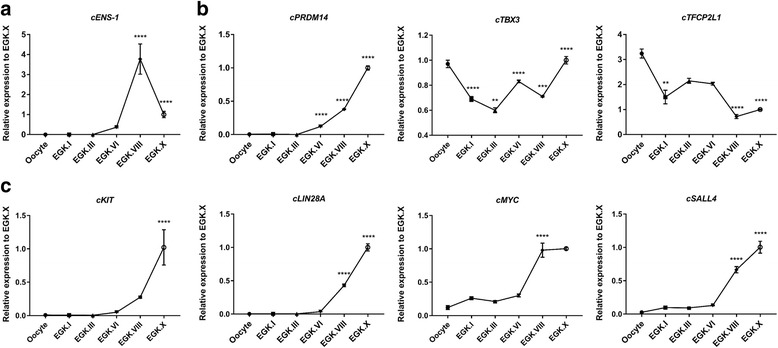
Pluripotency-related genes expression during the chicken developmental stage of pluripotency acquisition. **a** Chicken embryonic stem cell-related gene *cENS-1*, **b** naive pluripotency-related genes *cTFCP2L1*, *cTBX3*, and *cPRDM14*, and **c** general pluripotency-related genes *cKIT*, *cLIN28A*, *cMYC*, and *cSALL4* expression in oocyte and intrauterine chicken embryos were analyzed using qRT-PCR. *cGAPDH* was used as a reference gene. Results are shown as mean ± standard error of mean (n = 3). Significant differences of the relative gene expression between consecutive developmental stages (Oocyte vs. EGK.I, EGK.I vs. EGK.III, EGK.III vs. EGK.VI, EGK.VI vs. EGK.VIII, and EGK.VIII vs. EGK.X) were represented as ** *P* < 0.01, *** *P* < 0.001 and **** *P* < 0.0001

To further investigate the developmental stage in the acquisition of pluripotency and identify the factors involved in the chicken pluripotency network, we examined the comprehensive pluripotency-related marker expression by qRT-PCR across developmental stages from the oocyte to the EGK.X embryo. First, we examined the expression profile of *cENS-1*, which is a restrictive chicken endogenous retrovirus-like sequence and embryonic stem cell marker [28]. We found that *cENS-1* is significantly upregulated between EGK.VI and VIII but its expression is significantly downregulated between EGK.VIII and X, which is similar to the expression of *cNANOG* during developmental stages (Fig. 5a). Next, we investigated the expression of naive pluripotency-related markers, including *cTFCP2L1*, *cTBX3*, and *cPRDM14*, and also general pluripotency-related markers, including *cKIT*, *cLIN28A*, *cMYC*, and *cSALL4* [2, 29, 30]. These genes have been mainly defined in the mammalian species, however their role on the pluripotency acquisition during chicken intrauterine development is not clear. In our results, the naive pluripotency markers *cTFCP2L1* and *cTBX3* were found to be maternally inherited genes, and expression of the *cPRDM14* gene was gradually upregulated from EGK.VI until EGK.X (Fig. 5b). The general pluripotency markers, including *cLIN28A*, *cMYC*, and *cSALL4* were significantly upregulated between EGK.VI and VIII, and the expression of *cKIT* was upregulated between EGK.VIII and X (Fig. 5c). Taken together, these results suggest that the acquisition of pluripotency during chick embryonic development occurs at around stage EGK.VI to EGK.VIII.

## Discussion

The transition to the pluripotent state from the totipotent state in embryonic development is necessary for ZGA, cell cleavage, and regulation of cell fate [31–33]. In early embryogenesis, several transcription factors play a pivotal role in pluripotent acquisition and maintenance during embryogenesis. To date, the mechanisms of acquisition of pluripotency have been intensively studied in mammals and several vertebrates in vivo and in vitro. Regulation of *Nanog* is important for early development and the acquisition of pluripotency in the epiblast in mammals [34]. *Pou5f1* is expressed as early as the preimplantation embryo in the mouse [34], and also the mouse pluripotent embryonic stem cells (ESCs) are controlled by *Pou5f1*. Another core transcription factor, *Sox2*, which belongs to the *SoxB1* subfamily of genes [35], is also essential for the maintenance of the undifferentiated state in ESCs [36, 37].

In this regards, transcriptional factors, including *Nanog*, *Pou5f1* and *Sox2* play pivotal role in core regulatory network of pluripotency. Our results showed that the protein alignment of these core pluripotent transcription factors in chicken with other vertebrates revealed that they are fairly well conserved in the protein sequence among vertebrates. In particular, SOX2 has a sequence similarity of more than 90% in human, mouse, chicken, zebrafish, and African clawed frog. This may imply that these core transcriptional factors share the similar role in the pluripotency network among vertebrates. Although there are only a few studies, it has been recently reported that pluripotency seems to be evolutionarily conserved among amniotes, and mammalian core transcriptional factors could reprogram non-mammalian somatic cells into pluripotent stem cells [30, 38, 39].

However, the core pluripotent transcription factors that govern the acquisition of pluripotency during pre-ovipositional development have yet to be investigated in birds. Although both *Pou5f1* and *Sox2* are maternally inherited transcripts in mouse and they induce *Nanog* expression before ZGA [40], according to our results, the chicken seems to have a distinctive process for the acquisition of pluripotency. In this report, *cSOX2* is not expressed during folliculogenesis and intrauterine embryonic development, but detected in the embryo after oviposition, indicating that it is not involved in the initial acquisition of pluripotency. This is consistent with a previous report that *cSOX2* in the oviposited chicken embryo is first detected from HH stage 4 as the earliest pan-neural marker in the specified neuroectoderm [41, 42]. Thus, unlike in mammals, *cSOX2* in chicken seems to only be involved in early neural specification without a role in pluripotency networks. In the case of lower vertebrates, *Sox19b* is maternally inherited in *Danio rerio* among the *SoxB1* family and plays an important role in the acquisition of pluripotency, whereas *Sox3* carries out such a maternal contribution in *Xenopus laevis* [10, 43]. In avian species, it was recently reported that both finch and chick blastoderms at oviposition remarkably expressed *SOX3* [30], which is also known to be expressed in epiblast precursors [42]. Since the SOXB1 factors share more than 90% amino acid identity in the DNA binding HMG box region for transcriptional activation [35], acquisition of pluripotency in avian species may be regulated by another chicken *SOXB1* family member instead of *cSOX2*. Our results show that *cSOX3* may play important role in pluripotency network instead of *cSOX2* in avian species. It has been reported that mammalian SOX3 can replace the function of SOX2 during the reprogramming process, and SOX3 can compensate the absence of SOX2 to maintain the pluripotency and self-renew of ESC [44–47]. Similar to the pattern of *cNANOG* expression, intriguingly, maternally inherited *cSOX3* is upregulated between EGK.III and EGK.VI, indicating that *cSOX3* may involve in the initial establishment of pluripotency network in chicken embryos. Accordingly, further detailed investigation is required to determine how SOX3 is involved and regulated in the pluripotency network in avian species.

Furthermore, *cNANOG* transcripts were detected from between the white follicle and oocyte stages, indicating that among the core transcriptional factors, *cNANOG* is maternally inherited in embryos. *cNANOG* transcripts were weakly expressed compared with the EGK.X embryo, but its expression was dramatically increased between EGK.V and VI and localized in a heterogeneous manner in the central region of chick embryos*. cPOUV* was not detected until EGK. VI, however, its expression was dramatically increased between EGK VI and VIII in this study. Therefore, *cNANOG* and *cSOX3* seem to be regulated independently from *cPOUV* and play an important role in the initial pluripotency network prior to *cPOUV* during early embryonic development in chicken.

To understand a comprehensive pluripotency network during chick early embryonic development, we compared the relative expression of pluripotency-related genes in embryos from oocyte to EGK.X. Among the naive pluripotency markers, including *cTFCP2L1*, *cPRDM14*, and *cTBX3* [13, 48, 49], both *cTFCP2L1* and *cTBX3* seem to be maternally supplied while expression of *cPRDM14* was significantly increased between EGK.III and VI. It is known that TFCP2L upregulates NANOG via LIF-independent pathways and TBX3 is directly bound at NANOG and functions in upregulation of NANOG in mammals [50, 51]. In this regard, maternally inherited *cTFCP2L1* and *cTBX3* may regulate the initial upregulation of *cNANOG* or may be involved in the initial acquisition of pluripotency in chicken. Meanwhile, chicken ESC marker *cENS-1* and the general pluripotency markers *cKIT* and *cLIN28A* were gradually upregulated from EGK.VI until EGK.X, whereas *cMYC* and *cSALL4* seem to be maternally inherited but also dramatically upregulated between EGK.VI and VIII. Taken together, most of the pluripotency-associated genes were remarkably upregulated between EGK.VI and VIII, indicating that acquisition of the pluripotency network in the chicken embryo may be established between EGK.VI and VIII.

Meanwhile, it has been reported that the ZGA may be accompanied by acquisition of pluripotency via transcriptional factors in vertebrates [9, 52, 53]. Especially, in the frogs and zebrafish, acquisition of pluripotency is associated with ZGA, whereas acquisition of pluripotency occurred after ZGA in mice [39]. Although ZGA in avian species has not been identified yet, it was reported that the RNA polymerase II started to be activated during the late EGK.II to early EGK.III in chicken [16]. Accordingly, the understanding of the acquisition of pluripotency association with ZGA and the intricate molecular mechanisms of pluripotency regulating chicken embryo development requires further investigation.

## Conclusion

In conclusion, we found that among the core pluripotent transcription factors, *cNANOG* was maternally inherited and continuously expressed, but *cPOUV* was significantly upregulated between EGK.VI and VIII, and *cSOX3* instead of *cSOX2* was maternally inherited and continuously detected during intrauterine embryonic development in the chicken. Furthermore, we showed that the acquisition of pluripotency in the chick embryo may actively occurs at around stage EGK.VI to EGK.VIII, and birds seem to have a distinct regulatory mechanism of pluripotency compared with other vertebrates. Further studies should focus on the detailed mechanism of the pluripotency network via functional validation of transcriptional factors during early development in avian species from an evo-devo perspective.

## Abbreviations

CEF: Chicken embryonic fibroblast
EGK: Eyal-Giladi and Kochav stage
ENS-1: Embryonic normal stem cell 1
GADPH: Glyceraldehyde 3-phosphate dehydrogenase
HH: Hamburger Hamilton stage
HMG: High mobility group
iPSC: Induced pluripotent stem cell
KIT: KIT proto-oncogene receptor tyrosine kinase
LIN28A: Lin-28 homolog A
MYC: MYC proto-oncogene bHLH transcription factor
NANOG: Nanog homeobox
NCBI: National Center for Biotechnology Information
PGC: Primordial germ cell
Pou5f1: POU domain class 5 transcription factor 1
Pou5f3: POU domain class 5 transcription factor 3
POUV: POU domain class 5 transcription factor 3
PRDM14: PR/SET domain 14
qRT-PCR: Quantitative realtime-polymerase chain reaction
RT-PCR: Reverse transcription-polymerase chain reaction
SALL4: Spalt like transcription factor 4
SOX2: SRY-box 2
SOX3: SRY-box 3
TBX3: T-box 3
TFCP2L1: Transcription factor CP2-like 1
WF: Large white follicle
ZGA: Zygotic genome activation
